# Secondary Bacterial Infections in Mucormycosis-COVID-19 Cases: Experience during the Second COVID-19 Wave in India

**DOI:** 10.1128/spectrum.00919-22

**Published:** 2022-10-27

**Authors:** Neha Sharad, Smriti Srivastava, Parul Singh, Mamta Puraswani, Sharad Srivastav, Rajesh Malhotra, Anjan Trikha, Purva Mathur

**Affiliations:** a Department of Microbiology, AIIMS, New Delhi, India; b Department of Laboratory Medicine, JPNATC, AIIMS, New Delhi, India; c Department of Orthopaedic, AIIMS, New Delhi, India; d Department of Anaesthesia, AIIMS, New Delhi, India; Indian Institute of Science Bangalore

**Keywords:** COVID-19, mucormycosis, secondary infections

## Abstract

In the second wave of COVID-19 in India, there was a new challenge in the form of mucormycosis. Coinfection with mucormycosis was perilous as both conditions required a prolonged hospital stay, thus serving as an ideal platform for secondary infections. Using a retrospective observational study, we studied secondary infections and their impact on the outcome in COVID-19 patients with mucormycosis. The outcome in these patients was evaluated and compared with COVID-19 patients with mucormycosis but without any secondary infection. SPSS V-20 was used for data analysis. Fifty-five patients tested positive for mucormycosis (55/140; 39.28). Twelve out of these 55 (21.8%) developed secondary infections during their hospital stay. Bloodstream infection was the most common (42.86%) secondary infection. The Gram-negative (GN) organisms were more common (11/16; 68.75%) compared with the Gram-positives (GP) (5/16; 31.25%). But the most common isolate was Enterococcus faecium (5/16; 31.25%). A high percentage of microorganisms isolated were multidrug-resistant (15/16; 93.75%). Two out of five (40%) isolates of Enterococcus faecium were vancomycin-resistant (VRE). High resistance to carbapenems was noted in the GN isolates (9/11; 81.81%). The comparison of length of stay in both subgroups was statistically significant (*P* value <0.001). When compared, the length of stay in people with adverse outcomes was also statistically significant (*P* value <0.001). Procalcitonin (PCT) had a positive predictive value for the development of secondary bacterial infections (*P* value <0.001). Antimicrobial stewardship and strict infection control practices are the need of the hour.

**IMPORTANCE** Although our knowledge about COVID-19 and secondary infections in patients is increasing daily, little is known about the secondary infections in COVID-19-mucormycosis patients. Thus, we have intended to share our experience regarding this subgroup. The importance of this study is that it brings to light the type of secondary infections seen in COVID-19-mucormycosis patients. These secondary infections were partially responsible for the mortality and morbidity of the unfortunate ones. We, as health care workers, can learn the lesson and disseminate the knowledge so that in similar situations, health care workers, even in other parts of the world, know what to expect.

## INTRODUCTION

Since the pandemic began, the world has come to a standstill and is still reeling under the consequences. Globally, as of March 9, 2022, there have been 448,313,293 confirmed cases of COVID-19, including 6,011,482 deaths (1). However, not all deaths can be attributed primarily to SARS-CoV-2; COVID-19 has been known to cause many symptoms and complications that, along with secondary infections, have contributed significantly to this number (2, 3).

Hospital beds were scarce during the second wave of COVID-19 in India due to a sudden enormous surge of cases. Along with an increase in the number in severe cases, we had a new challenge in the form of mucormycosis. It was declared a notifiable disease on May 19, 2021, by the Central government of India under the Epidemic Diseases Act of 1897. Mucormycosis rarely causes infection in immunocompetent hosts. In susceptible hosts, it causes an angio-invasive, rapidly progressing, potentially life-threatening condition requiring emergency and aggressive management to minimize morbidity and avoid mortality.

The immune system in COVID-19-positive patients is dysregulated with severe inflammation, requiring immune-suppressant drugs, including corticosteroids, to manage the infection, which prevents the phagocytic cells of our immune system from attacking the fungus. Diabetes mellitus is a common and well-known risk factor for mucormycosis. COVID-19 is known to cause a state of iron overload and is associated with high blood sugar levels. All these factors may presumably be responsible for the surge in mucormycosis (4, 5). COVID-19 with mucormycosis is double trouble, requiring extended hospital stays. This setting provides an ideal platform for secondary infections. We witnessed a sudden surge of cases during India's second wave of COVID-19 (from the end of March 2021 until June 2021). Concomitantly, there was a massive surge of COVID-19 patients that had mucormycosis. This study reports the profile of secondary bacterial infections in COVID-19 cases with mucormycosis.

## RESULTS

**FIG 1:**
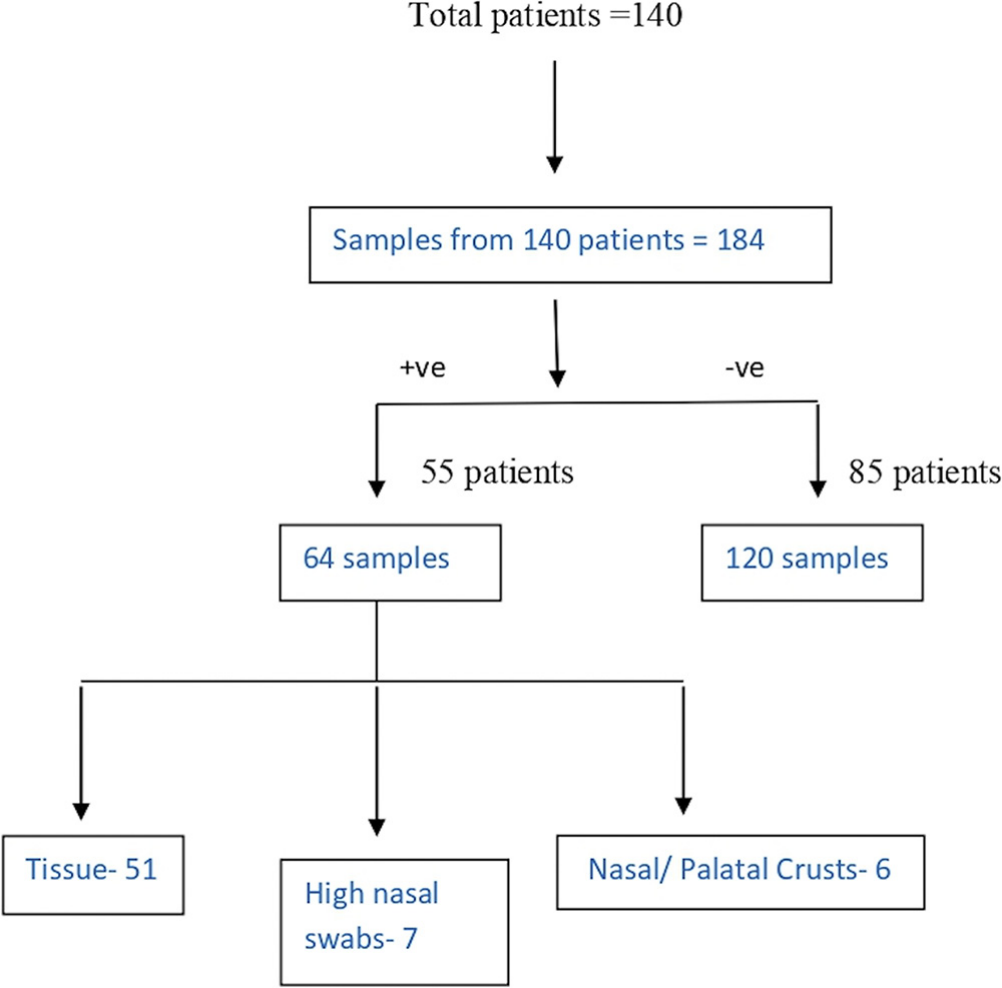
Flowchart depicting the samples received from suspected mucormycosis patients.

**FIG 2:**
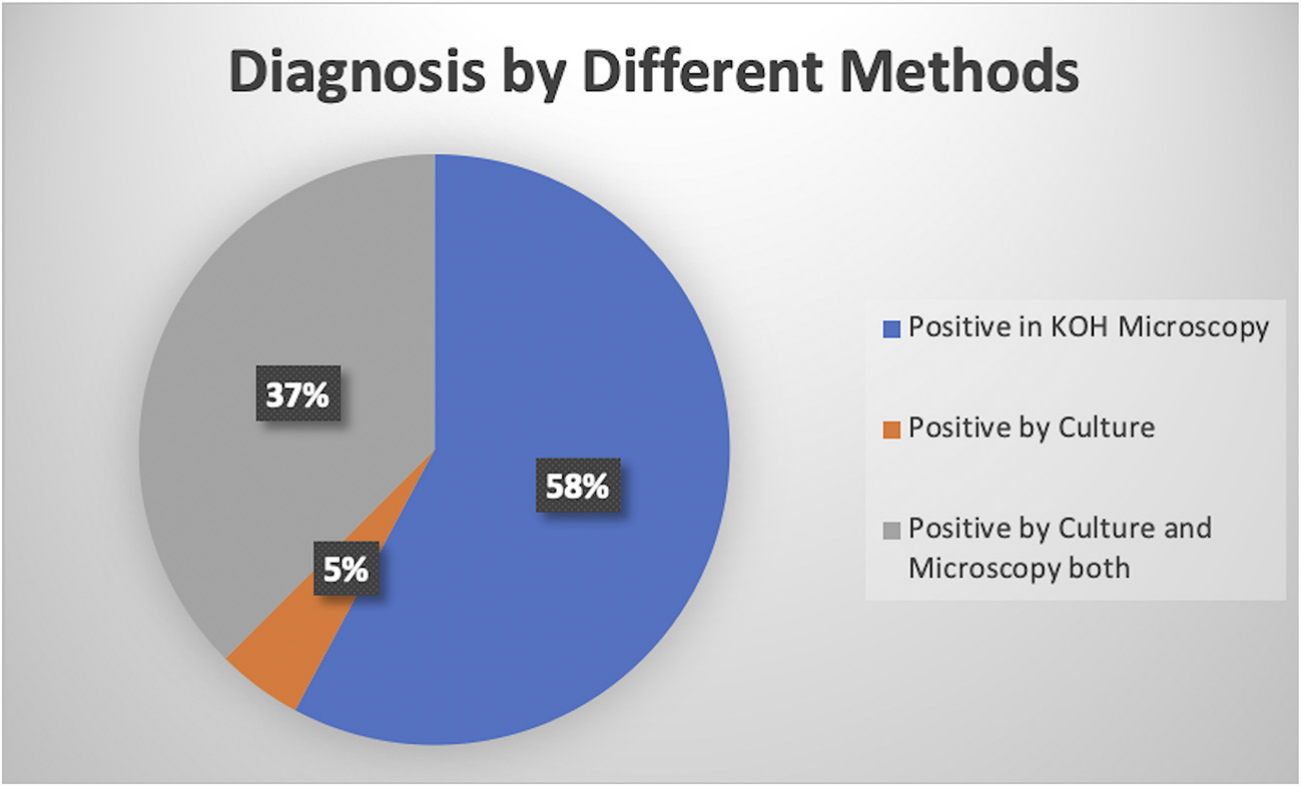
Mucormycosis diagnosis by various methods.

**FIG 3:**
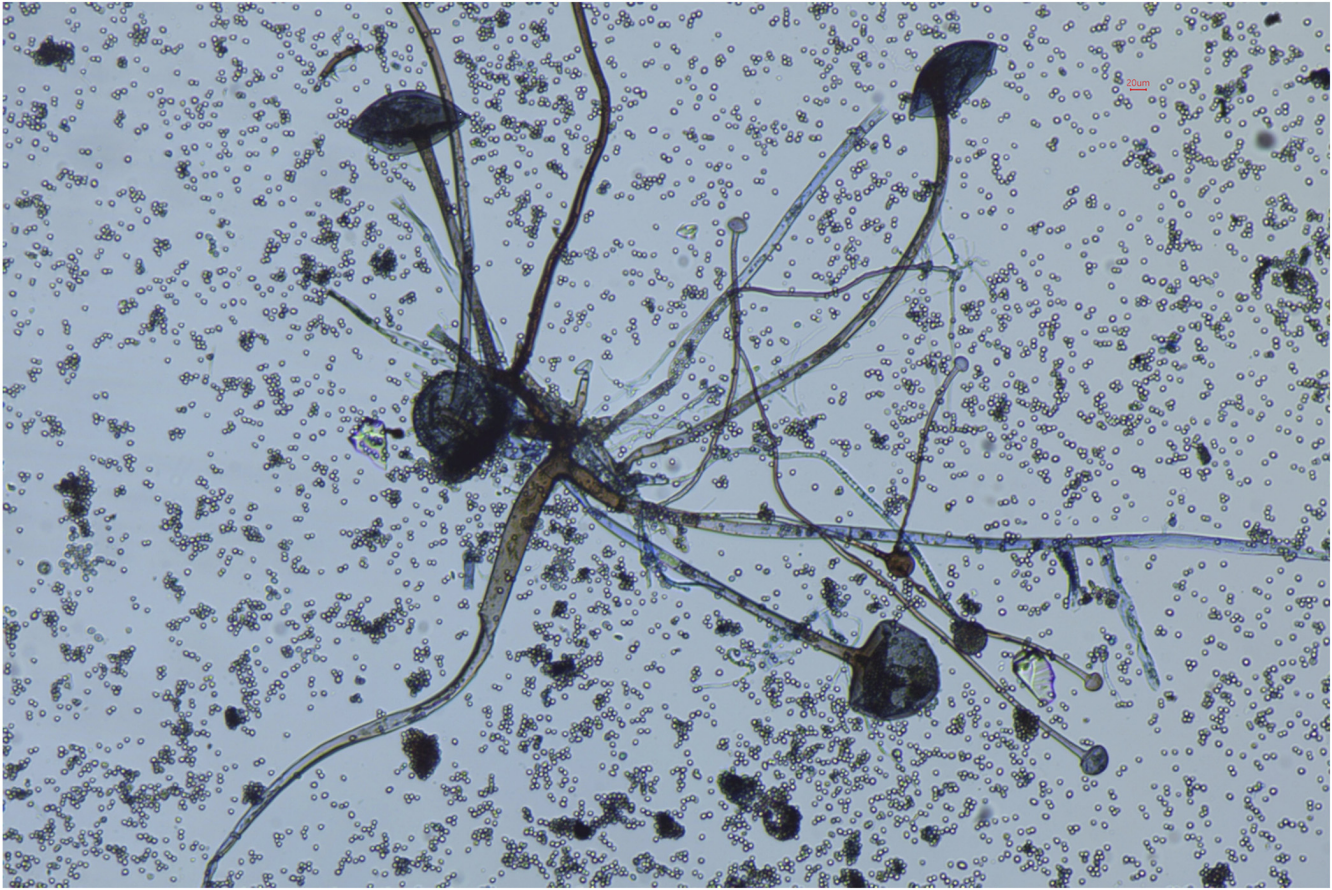
Rhizopus arrhizus seen in LPCB Mount, total magnification ×100.

In our study, 184 samples were received in our laboratory from patients suspected to have mucormycosis, details in Table 1 and Fig. 1. Of these, 64 samples from 55 patients were positive for mucormycosis, and these 55 people were included in our study (nine were repeat samples and therefore excluded). Of these, 37 were positive by potassium hydroxide (KOH), three by culture only, and 24 by KOH and culture (Fig. 2). The culture was positive in 42.18% (*n* = 27) cases. Lactophenol cotton blue staining of the teased growth from positive cultures was done to aid identification (Fig. 3).

**TABLE 1:** Sample wise distribution for mucormycosis

Sample type	Total samples	Positive samples
Tissue/turbinate biopsy	103	51
High nasal swabs	47	7
Nasal/palatal crusts	31	6
Sputum	3	0
Total	184	64

Patients with mucormycosis were aged from 28 to 76 years (median age, 51.6 years). The gender-based distribution was 4.6:1, with males being predominant. All but three samples were from suspected cases of rhino-orbital mucormycosis; three samples were from one single patient of suspected pulmonary mucormycosis.

The most common species identified in our set-up was *Rhizopus* (25/27; 92.6%), followed by one case of each *Rhizomucor* (1/27; 3.7%) and *Mucor* (1/27; 3.7%) species. The most common isolate identified as Rhizopus arrhizus (20/27; 74.07%).

These 55 patients who tested positive for mucormycosis were evaluated further for secondary infection. All of the 55 patients were known to have diabetes mellitus and received corticosteroids to manage their COVID-19 infection.

### Secondary bacterial infections.

**FIG 4:**
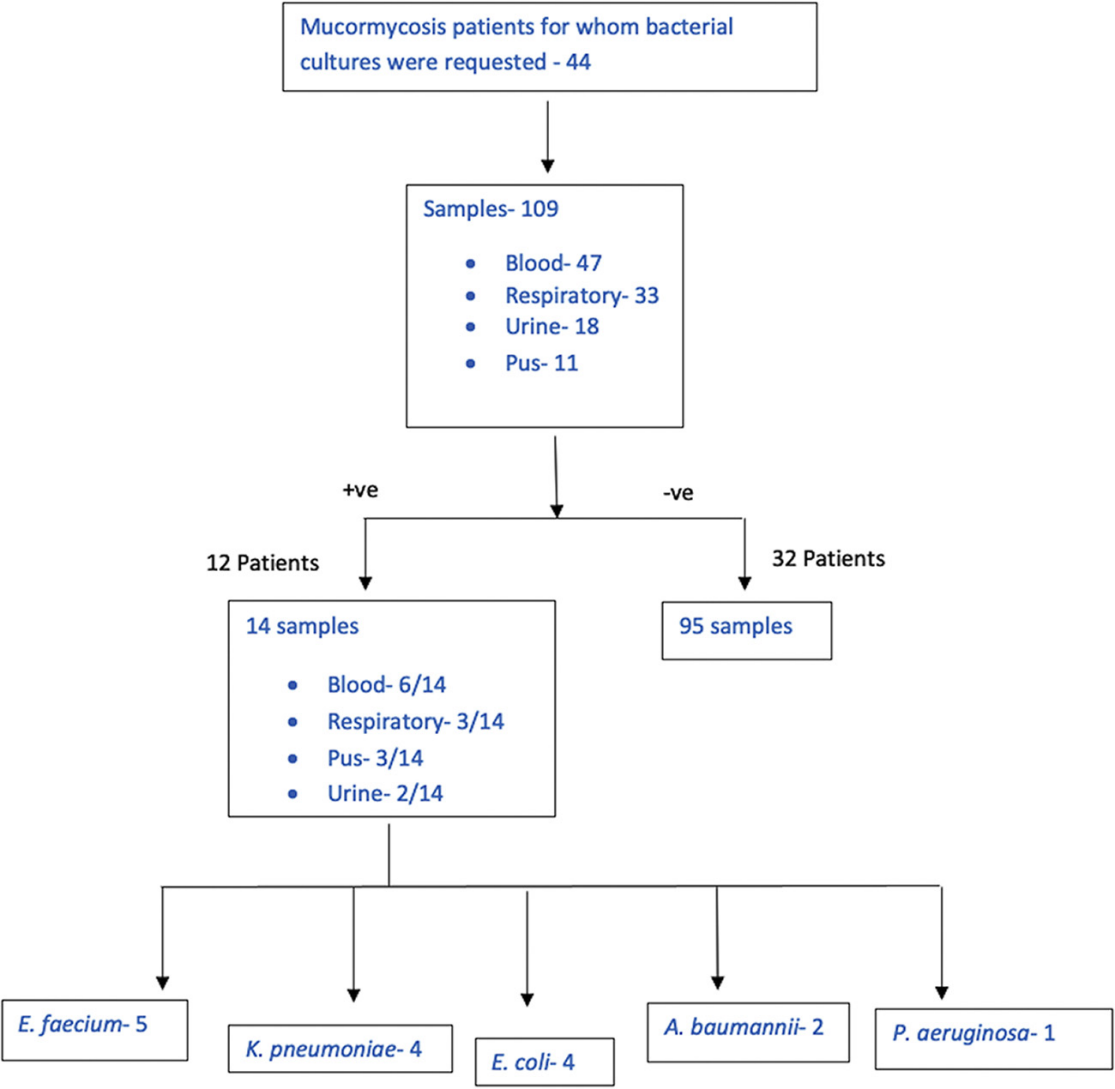
Flowchart depicting the samples received for bacterial culture from patients of mucormycosis.

A total of 109 bacterial culture samples from 44 patients were received in our lab for microbiological evaluation. Fourteen samples from 12 patients were positive for bacterial pathogens. See Fig. 4 for details.

Twelve out of the total 55 (21.8%) people with mucormycosis developed secondary infections during their stay in the hospital. Six out of these 14 positive cultures were of blood (6/14; 42.86%), followed by respiratory (3/14; 21.43%), pus (3/14; 21.43%), and urine (2/14; 14.29%) (Table 2). The mean age was 48.1 (range 27 to 74 years). Seven were males and five were females. Three patients had a polymicrobial infection; details are given in Table 3. Among the polymicrobial infections, two were bloodstream infections, and the third was pus samples from a surgical debridement wound. E. faecium was isolated from all three samples of polymicrobial infection. E. faecium was isolated along with *Candida rugosa* in one of these polymicrobial bloodstream infections.

**TABLE 2:** Sample wise distribution for secondary bacterial infections

Sample type	Total samples	Positive samples
Blood	47	6
Respiratory	33	3
Pus	11	3
Urine	18	2
Total	109	14

**TABLE 3:** Polymicrobial infections

Patient ID	Patient details	Sample	Organisms	Outcome
Patient 7	Female/33	Blood	E. faecium P. aeruginosa	Death
Patient 10	Male/35	Pus	E. faecium K. pneumoniae	Recovered
Patient 11	Male/40	Blood	E. faecium *C. rugosa*	Death

One patient had two different infections during his hospital stay, bloodstream and respiratory, different microorganisms isolated from both the samples. Thus, we identified 16 bacterial isolates, details given in Tables 4 and 5.

**TABLE 4:** Gram-negative organism with antimicrobial susceptibility pattern and patient profile

Patient number	Patient profile	Sample	Organism	OutcomE	Imipenem	Meropenem	Tigecycline	Minocycline	Aztreonam	Ceftazidime-avibactam	Piperacillin-tazobactam	Gentamicin	Amikacin	Levofloxacin	netilmicin	Ceftriaxone	Cefoperazone-sulbactm
Patient 1	Female/42	Blood	E. coli	Recovered	I	S	S	S	R	S	R	R	S	R	S	R	R
Patient 2	Male/53	Tracheal aspirate	E. coli	Recovered	R	I	S	S	R	R	R	S	S	R	R	R	I
Patient 3	Female/74	Blood	A. baumannii	Death	R	R	S	S	R	R	R	R	R	R	R	R	R
Patient 4	Male/52	Tracheal aspirate	E. coli	Recovered	R	R	S	S	R	R	R	R	R	R	R	R	R
Patient 5	Male/50	Blood	A. baumannii	Death	R	R	S	S	R	R	R	R	R	R	R	R	R
		Tracheal	E. coli		R	R	S	S	R	R	R	S	S	R	R	R	R
Patient 6	M/47	Pus	K. pneumoniae	Recovered	R	R	R	R	R	R	R	R	R	R	R	R	R
Patient 7	Female/33	Blood	P. aeruginosa	Death	R	R	-^*a*^	-	R	S	R	S	S	R	S	-	I
Patient 8	Female/59	Blood	K. pneumoniae	Death	R	R	R	R	R	R	R	R	R	R	R	R	R
Patient 9	Male/65	Pus	K. pneumoniae	Recovered	S	S	S	S	R	S	I	I	S	S	S	R	S
Patient 10	Male/35	Pus	K. pneumoniae	Recovered	R	R	R	I	R	R	R	R	R	R	R	R	R

a -, Not tested.

**TABLE 5:** Gram-positive organisms with antimicrobial susceptibility pattern and patient profile

Patient number	Patient profile	Sample	Organism	Outcome	Vancomycin	Penicillin	HLA	Linezolid	Tetracycline	Teicoplanin	Ciprofloxacin	Erythromycin	Nitrofurantoin
Patient 11^*c*^	Male/40	Blood	E. faecium	Death	R	R	S	S	S	S	R	R	-^*d*^
		Urine			R	R	S	S	S	S	R	R	I
Patient 12	Female/27	Urine	E. faecium	Recovered	S	R	S	S	S	S	R	R	R
Patient 7^*a*^	Female/33	Blood	E. faecium	Death	S	S	S	S	S	S	S	R	-
Patient 10^*b*^	Male/35	Pus	E. faecium	Recovered	R	R	R	S	S	R	R	R	-

a Patient 7, same patient, two isolates from one blood sample.

b Patient 10, same patient, 2 isolates from one pus sample.

c Patient 11, patient had E. faecium from two samples, blood and urine.

d -, Not tested.

Overall, Gram-negative (GN) organisms were more common causative agents (11/16; 68.75%) in comparison with Gram-positives (GP) (5/16; 31.25%). However, the most common organism isolated was Enterococcus faecium (5/16; 31.25%), two from blood, two from urinary samples, and one from pus (Table 1). The following common organisms were Klebsiella pneumonia (4/16; 25%), one from blood, three from pus; and Escherichia coli (4/16; 25%), one from blood, two from respiratory samples, and one from pus. Fifteen out of the total 16 isolates were multidrug resistant (MDR).

Acinetobacter baumannii and E. faecium were the most common isolates in bloodstream infections, two out of seven.

### Gram-negative organisms and their susceptibility profile.

Eleven out of 16 isolates were GN, the most common being *K. pneumonia* (*n* = 4) along with E. coli (*n* = 4), A. baumannii (*n* = 2), and Pseudomonas aeruginosa (*n* = 1). All 11 GN isolates were resistant to ceftriaxone and aztreonam and sensitive to colistin. High resistance to carbapenems was noted, with nine out of 11 isolates (81.81%) resistant. Three out of four isolates of K. pneumoniae were resistant to carbapenem (75%). Both the isolates of A. baumannii were resistant to carbapenems (100%), and the single isolate of Pseudomonas aeruginosa was also resistant to carbapenems. Three out of 11 GN strains were resistant to tigecycline, all three being carbapenem-resistant K. pneumoniae (CRKP).

The fatal outcomes seen in this subgroup were all infected with carbapenem-resistant strains. Two patients with *CRKP*, one with carbapenem-resistant A. baumannii (CRAB) and one who had a polymicrobial infection, were infected with carbapenem-resistant P. aeruginosa (CRPA) along with vancomycin-sensitive E. faecium (patients 3, 5, 7, 8 in Tables 4 and 6).

**TABLE 6:** Antimicrobial susceptibility of GN isolates

Antibiotics	K. pneumoniae (4)	E. coli (4)	A. baumannii (2)	P. aeruginosa (1)	Total (11)
Imipenem	1	-^*a*^	-	-	1 (9.09%)
Meropenem	1	1	-	-	2 (18.18%)
Tigecycline	1	4	2	1	8 (72.72%)
Colistin	4	4	2	1	11 (100%)
Aztreonam	-	-	-	-	0 (0%)
Ceftazidime-avibactam	1	1	-	1	3 (27.27%)
Piperacillin-tazobactam	-	-	-	-	0 (0%)
Gentamicin	-	2	-	1	3 (27.27%)
Amikacin	1	3	-	1	5 (45.45%)
Levofloxacin	1	-	-	-	1 (9.09%)
Ceftriaxone	-	-	-	-	0 (0%)
Cefoperazone-sulbactam	1	-	-	-	1 (9.09%)

a -, Not tested.

### Gram-positive isolates and their susceptibility profile.

All five isolates of the GP organism were E. faecium. Four of these five isolates were MDR, and two out of them were vancomycin-resistant (details of patients in Table 5). All five isolates were sensitive to linezolid and tigecycline. Three isolates were susceptible to high-level aminoglycosides. One isolate was susceptible to all drugs except erythromycin, isolated from bloodstream infection from a 33-year-old female, along with P. aeruginosa*;* this patient succumbed while in intensive care units (ICUs). Two fatalities (2/5; 40%), patients 7 and 11, were seen in patients infected with *Enterococci* (Table 5).

### Mucormycosis patients with and without secondary infection.

The mean age of the people who developed secondary infection was 48.1, and 50.7 for those who did not develop a secondary infection (*P* value 0.570). Gender-wise distribution (male: female) was 1.4:1 and 5.33:1 for the two groups, respectively (*P* value 0.145).

Twelve out of 55 (21.8%) patients with mucormycosis developed secondary infections after an average length of stay of 38.33 days. Meanwhile, 43 (78.1%) patients with mucormycosis did not develop secondary infections; their average length of stay being 15.12 days (*P* value <0.001). The average duration of stay in patients developing secondary infection was more than twice that of those not developing a secondary infection and was statistically significant (Table 7).

**TABLE 7:** Mucormycosis patients with and without secondary infection

Patient parameter	With secondary infection (*n* = 12)	Without secondary infection (*n* = 43)	*P* value^*a*^
Mean age	48.1 +/– 14.04	50.7 +/– 13.9	0.570
Gender (M:F)	1.4:1 (M = 7, F = 5)	5.33:1 (M = 34, F = 9)	0.145
Mortality	5/12 (41.67%)	8/43 (18.6%)	0.096
Length of stay in patients who died	21.33 +/– 15.38	9.12 +/+ 5.96	**<0.001**
Length of stay (Days)	38.33 +/– 14.94	15.8 +/– 9.83	**<0.001**
PCT	24.4 ng/mL (+/– 30.77)	0.28 ng/mL (+/− 0.34)	**<0.001**
CRP	242.04 +/– 176.34	184.89 +/– 135.41	0.232

a Values in bold are statistically significant *P*-values.

Five out of 12 (41.67%) patients who had developed a secondary infection succumbed after an average length of stay of 21.33 days, the most common cause of death being a septic shock. Eight out of 43 (18.6%) without any secondary infection succumbed, at an average stay of 9.12 days in the hospital (*P* value <0.001). The cause of death was multifactorial, including cardiac arrest, severe refractory COVID-19 acute respiratory distress syndrome, and severe metabolic acidosis.

### Viral markers.

We received samples of viral markers from nine patients for hepatitis B surface antigen (HbsAg) and antibodies for hepatitis C and HIV. All patients were nonreactive for all viral markers. Only one patient among the mucormycosis-positive patients was a case of chronic hepatitis B, not on medication and not tested in our laboratory (patient 1). She subsequently developed secondary bloodstream infection by MDR E. coli. Despite having multiple comorbidities, she was discharged after successfully recovering.

### Biomarkers.

We evaluated the procalcitonin (PCT) and C-reactive protein (CRP) values in patients with secondary infection. The sampling date was the same as the microbiological sample date; the mean PCT was 24.4 ng/mL, and 242.04 was the mean CRP value. Mean procalcitonin levels in patients with bloodstream infection was 43.13 ng/mL. PCT in patients without any secondary infection was 0.28 ng/mL, whereas the levels in patients developing secondary infection were 24.4 ng/mL with the onset of secondary infection (*P* value <0.001). Prior to developing secondary infection, the mean PCT was <0.15 ng/mL which increased to 24.4 ng/mL afterwards (*P* value <0.001). CRP levels in patients without secondary infection were 184.89, and in patients with secondary infection were 242.04. CRP was constantly elevated. No significant difference was noted between both groups (*P* value 0.232) (please see Table 7).

## DISCUSSION

Rhino-orbital is the most common type of mucormycosis worldwide (6). In our study, 54 patients were cases of rhino-orbital mucormycosis, and one patient had pulmonary mucormycosis. *Rhizopus* species is the predominant agent causing mucormycosis, with *Rhizopus arrihzus* being the most common species isolated (7). Our findings are similar, with *Rhizopus arrihzus* being isolated in 74.07% of culture-positive cases.

Studies have shown a high association between mucormycosis developing in COVID-19 patients and diabetes mellitus and corticosteroids (6–9). In our study, all infected patients were known cases of diabetes mellitus and had a history of corticosteroid usage during the management of COVID-19 infection.

Mucormycosis is a life-threatening disease requiring urgent management, so a positive KOH mount in a suspected case is a go-ahead sign for managing these patients. Culture is less sensitive; positivity is seen in about 40% of cases (10). Thus, waiting for culture reports in these patients is not advisable. In our study, culture positivity was 42.18%, similar to Walsh et al. (10).

Data on the secondary infection in COVID-19 patients with mucormycosis is lacking. Thus, we intend to share our experience regarding this subgroup. Considerable data on secondary infections in COVID-19 patients without mucormycosis has been published, and we have compared our findings with three such studies (Table 8). The incidence of secondary infections in these patients has been reported to be 3.6% to 14% (11–13). In our study, we have reported 21.81% of secondary infections in COVID-19-positive patients with mucormycosis. Our study's most common type of secondary infection was bloodstream infection; two studies from India, mentioned in Table 8, Vijay et al. and Khurana et al., have reported similar findings. Another study, Li et al., from China has reported pulmonary infections as the most common secondary infection (11–13).

**TABLE 8:** Studies: Secondary bacterial infections in COVID-19 patients^*a*^

Study	Vijay et al.	Khurana et al.	Li et al.
Single/multicentre study	Multi	Single	Single
Country	India	India	China
Most common organism	K. pneumoniae A. baumannii	K. pneumoniae A. baumannii	A. baumannii K. pneumoniae
Incidence of SBI	3.6%	14%	6.8%
% MDRO	47.1%	84%	85.8%
SBI	Bloodstream, **49.88%** Respiratory, 39.77% Urinary, 10.33%	Bloodstream, **42.8%** Respiratory, 18.6% Urinary, 21.8%	Bloodstream- 27.04% Respiratory, **69%** Urinary, 3.77%
Reference no.	11	12	13

a Bold denotes most common SBI in the respective study.

Mortality seen in our patients developing secondary infection was 41.67%, less than that reported by Ji et al. (49%) but more in comparison to the first wave mortality, reported from our center by Khurana et al. (33%) (12, 13).

In two studies from India mentioned in Table 8, the most common organisms were *K. pneumonia* and A. baumannii. In both studies, GN infections were more common than the GP. In a study from our center from the first COVID-19 wave by Khurana et al., GN isolates were predominant, with no cases of *Enterococcus* species reported (11, 12).

In patients who are critical and admitted to ICUs, sometimes it is challenging to differentiate COVID-19 acute respiratory distress syndrome from ventilator-associated pneumonia. So, these patients receive antimicrobials targeting the GN, the usual pathogen. This could select the GP, including the *Enterococcus* species, thus explaining the increase in cases. The high incidence of E. faecium could be due to extended hospital stays in an ICU setting, and exposure to broad-spectrum antibiotics such as cephalosporins and carbapenems (14–16). In a study by Harthug et al., cephalosporins use and extended hospital stay were predisposing factors for enterococcal infections (16). All patients with E. faecium were young (mean age 35) and critical, admitted to ICU and high dependency units. A fatal outcome was seen in two people with bloodstream infections infected with multidrug-resistant E. faecium*. Enterococcus* species are notorious for causing resistance due to the transfer of resistance-causing genes through plasmid and are associated with higher mortality.

We noted a high proportion of MDR isolate (93.75%) associated with COVID-19, slightly higher than in studies quoted in Table 8, 47.1%, 84%, and 85.8% (11–13). The reasons may include prolonged hospital stays and ICU admissions. These events trigger widespread and injudicious use of antibiotics, and the vicious cycle of antimicrobial usage and emergence of resistant strains continues.

Emerging data show that more than 70% to 90% of COVID-19 patients received antibacterial drugs against only 10% indicated (17, 18). This increase in antibiotic administration can cause a strong selective pressure on bacterial pathogens to evolve their resistance leading to the increased incidence of drug-resistant bacterial infections in the years after the COVID-19 pandemic.

Carbapenems are the first-line therapy for MDR GN infections. However, an increase in carbapenem-resistant (CR) organisms is being reported. Li et al. found 91.7% of CRAB and 76.7% of CRKP in their study from a COVID-19-designated hospital in China (13). It poses a more difficult challenge as the CR are resistant to β-lactam agents, thus limiting the treatment options.

The high prevalence of CR GN in COVID-19 patients could be due to various causes. Although personal protective equipment (PPEs) are a mandatory requirement while attending to people with COVID-19, their usage is primarily for the protection of health care workers. Change of PPEs between the handling of different patients is practically impossible. Isolation precautions for patients infected with CR strains could not be maintained due to a shortage of beds compared with the number of cases during the second wave in India. Also, the patients in our setting had mainly moderate to severe infections. The severity of infection could have prompted carbapenems for extended-spectrum beta-lactamases producing organisms, and prior carbapenem use is a risk factor for causing resistance (19, 20).

This rampant increase in CR strains poses a severe threat. Only tigecycline and colistin remain the options for treating these patients. However, a higher tigecycline resistance is also being reported from all around the world. In a study by Park et al., 37.8% of CRKP isolates were resistant to tigecycline (21). If this trend continues, we will be ushered toward the preantibiotic era. All the hard work done with the aid of hospital infection control practices and antimicrobial stewardship programs will be a total waste.

PCT and CRP have proven helpful in lower respiratory tract infections and help to differentiate between pure viral or secondary infection. In a study by Pink et al., a significant increase in both PCT and CRP levels was observed in patients with a secondary bacterial infection (22). In our study, an increase in PCT is directly associated with culture positivity, with values as high as 97.07 ng/mL (mean value = 24.4 ng/mL). However, for CRP, it was consistently elevated in patients with COVID-19 infection, and there was no correlation between its value and secondary infections. Thus, as CRP is consistently elevated, it might not have a predictive value for bacterial infections in COVID-19. Nevertheless, PCT has a positive predictive value for secondary bacterial infections overall and in COVID-19 patients.

Serum PCT may help identify secondary infections in patients with COVID-19. In isolated COVID-19, as in other viral infections, PCT levels usually remain normal. The lack of a PCT rise in viral infections may be due to virus-stimulated production of interferon-γ by macrophages, which inhibits TNF-α in the immune response. PCT has emerged as a valuable tool to facilitate decisions about antibiotic therapy in lower respiratory tract infections (22, 23).

Length of stay in hospital is corelated with the development of secondary infection and mortality. The longer the duration of stay in the hospital, the longer the chance of developing secondary infections.

### Conclusion.

A high incidence of MDR organisms in the presence of COVID-19 and mucormycosis poses a challenge to treating physicians and is associated with higher mortality. Culture-based testing should be carried out before antimicrobials are initiated. Controlled use of antibiotics in synergy with periodic surveillance and better hand hygiene practices will prevent the dissemination of drug-resistant organisms. We can use PCT as a guiding tool for evaluating secondary infection in such scenarios. Liberal use of corticosteroids should be checked. Limitations of our study include a monocentric setting, a small sample size, and a retrospective design.

## MATERIALS AND METHODS

### Study setting and design.

A retrospective observational analysis was conducted in our center, part of a 2,500-bedded tertiary care center in north India. Ours has been a dedicated set-up for COVID-19 since April 2020, where moderate to severe cases are admitted. The center has 285 beds, including 70 ICUs and high dependency units (HDUs).

Ours was a 2-month retrospective study, from May 1, 2021 to June 30, 2021, where we have studied secondary infections and their impact on the outcome in COVID-19 patients with mucormycosis. All demographic details of patients were taken from the hospital information system.

### Laboratory techniques.

All samples from suspected cases of mucormycosis, including tissue biopsy samples, nasal/palatal crusts, and high nasal swabs, were evaluated by conventional methods. Microscopy was done using a 10% KOH mount, aided with fluorescent brightener calcofluor white. Cultures for mycological growth were done on two sets of Sabouraud's dextrose agar (SDA) at 37°C and 25°C, each group having three tubes (SDA without antibiotics, SDA with gentamicin, and SDA with actidione). Positive cultures were further identified with the help of their macroscopic and microscopic features (growth teased and stained with lactophenol cotton blue stain), as per standard mycological methods.

Secondary infections in mucormycosis patients were studied based on conventional culture, microbiological profile, and identification and antibiotic susceptibility profile were done by Vitek 2 (bioMérieux). Microbiological profiles of organisms obtained from various samples like blood, respiratory (bronchoalveolar lavage, endotracheal aspirate, and sputum), and urine samples were compared. Colistin susceptibility was evaluated by broth microdilution as per CLSI 2021 guidelines.

PCT and CRP tests, done as part of routine tests, were evaluated for the study. The outcome in these patients was assessed and compared with COVID-19 patients with mucormycosis but without any secondary infection.

### Statistical analysis.

The 95% confidence interval values at <0.05 were considered significant. The proportions of secondary infections across admission locations and outcomes were compared using the Chi-square test of independence. Data analysis was done using SPSS V-20. MS Excel 2007 (Microsoft Corporation) was used for descriptive analysis.

Ethical approval was taken from the institutes' Ethics Committee (Ref No.: IEC-571/06.08.2021).
